# Integrative transcriptomic and microbiome analyses reveal thermal adaptation mechanisms in green and red color morphs of *Myzus persicae* (Hemiptera: Aphididae)

**DOI:** 10.3389/finsc.2026.1780864

**Published:** 2026-04-17

**Authors:** Yan Pang, Yifan Wang, Quan Deng, Xinwei Wang, Jitao Wang, Wenxin Xue

**Affiliations:** 1 Key Laboratory of Tobacco Pest Monitoring Controlling & Integrated Management, Tobacco Research Institute of Chinese Academy of Agricultural Sciences, Qingdao, China; 2 Sichuan Liangshan Company, Xichang, China; 3 Anhui Industry Co., Ltd., Hefei, China

**Keywords:** aphids, color morph, global warming, heat stress, thermal adaptation

## Abstract

Under global warming, the frequency and severity of agricultural pest outbreaks have intensified, posing serious threats to agriculture. The green peach aphid (*Myzus persicae* (Hemiptera: Aphididae)), an important agricultural pest, exhibits green and red color morphs and differentiated thermal tolerance, yet the underlying molecular mechanisms remain unclear. In this study, based on transcriptome and 16S rDNA amplicon sequencing, we analyzed the gene expression patterns and microbial community dynamics of green and red morphs of *M. persicae* under high-temperature stresses (30 °C and 35 °C) and across different exposure durations, comparing their similarities and differences in heat-response processes. Principal component analysis of transcriptomic data indicated that temperature had a greater influence on the physiological responses of *M. persicae* than body color, with a more pronounced effect observed at 35 °C. Differential gene expression analysis revealed overlap in temperature-responsive genes but different response patterns between the two morphs, suggesting activation of divergent molecular response mechanisms. Genes encoding heat shock proteins, detoxification-related enzymes, ribosomal protein family and so on were significantly up-regulated under high temperature, with a more pronounced induction in the green morph, indicating morph-specific regulatory strategies in response to thermal stress. Moreover, 16S rDNA sequencing revealed that the primary symbiont *Buchnera* displayed different relative abundance trends in the green and red morphs, remaining relatively stable in the red morph but declining markedly in the green morph under heat stress, potentially associated with their variation in thermal tolerance. Collectively, this study elucidates the molecular responses and microbe-mediated regulatory mechanisms underlying thermal tolerance in green and red morphs of *M. persicae*, providing novel insights into the thermal adaptation of aphids and a theoretical basis for developing pest management strategies under global warming.

## 1 Introduction

Climate change, especially global warming, has emerged as a major driver of pest dynamics by influencing insect physiology, behavior, and distribution, ultimately intensifying pest outbreaks and threatening agricultural production (1, 2). Insects are highly sensitive to temperature changes due to their rapid reproduction, large population sizes, and strong adaptability. Heat stress profoundly influences insect growth, development, reproduction, and distribution, highlighting the importance of elucidating thermal adaptation mechanisms for predicting pest population dynamics and developing climate-resilient pest management strategies (3, 4).

To understand how these physiological responses are achieved, it is therefore essential to examine the molecular pathways that regulate cellular protection and stress adaptation. The responses of insects to heat stress involve physiological, molecular, and behavioral regulation, primarily mediated through heat shock proteins (HSPs) and related transcription factors that maintain cellular homeostasis and repair heat-induced damage (5). For example, *Lygus hesperus* (Hemiptera: Miridae) exhibits significant upregulation of *Hsp70*, *Hsp40*, and small *Hsp* genes under high temperature (6), while *Grapholita molesta* (Lepidoptera: Tortricidae) shows elevated expression of *Hsp90* and *Hsp70* family genes following heat stress (7). In addition, detoxification enzyme systems such as cytochrome P450 (CYP) and glutathione S-transferase (GST) are involved in oxidative stress responses induced by high temperature (8). These findings demonstrate that insect thermal tolerance is a complex process governed by the coordinated regulation of multiple genes.

Beyond host genes, symbiotic microorganisms play crucial roles in insect adaptation to environmental stress (9–11). Symbionts not only provide essential nutrients but also participate in metabolic, immune, and stress-resistance regulation (12). High temperature has been shown to affect both the abundance and function of insect symbionts. For instance, the male-killing *Wolbachia* (Rickettsiales: Alphaproteobacteria) in *Drosophila melanogaster* (Diptera: Drosophilidae) decreases significantly at 28 °C (13), and the copy number of *Buchnera* in *Aphis craccivora* (Hemiptera: Aphididae) drops sharply at 35 °C (14).

Crucially, microbial symbionts are not merely passive passengers but active mediators of host phenotypes, including stress responses. For instance, obligate symbionts like *Buchnera* are essential for provisioning nutrients, and their density and metabolic activity are highly thermally sensitive, directly affecting host fitness under heat stress (15). Furthermore, facultative symbionts such as Serratia symbiotica or Rickettsiella viridis have been implicated in conferring thermal protection, potentially through modulating the expression of host genes involved in heat shock response or by stabilizing cellular homeostasis (16, 17). Serratia symbiotica has been shown to enhance host heat tolerance by maintaining *Buchnera* density and upregulating fatty acid metabolism, thereby supporting cellular homeostasis under stress (15, 17). Similarly, infection with viral symbionts can upregulate heat shock protein genes (e.g., *Hsp70*, *Hsp90*) in aphids, leading to improved thermal tolerance and niche expansion (18). To date, however, a systematic investigation that simultaneously quantifies the shifts in both host gene expression and the dynamics of the entire microbial community (including both primary and secondary symbionts) under thermal stress across distinct color morphs remains lacking.

The green peach aphid (*Myzus persicae* (Hemiptera: Aphididae)) is a cosmopolitan, highly polyphagous pest with high insecticide resistance, feeding on plant phloem sap and transmits numerous plant viruses (19, 20). It displays a wide array of colour polymorphism ranging from green to brownish red, which not only represent phenotypic differences but are also closely related to physiological adaptability (21–23). Aphid coloration like in *Acyrthosiphon pisum* (Hemiptera: Fabaceae) is likely determined by the composition of carotenoid compounds, with green morphs containing only yellow carotenoids, whereas red morphs possess additional red carotenoids (24). In addition, aphids provide a stable habitat and nutrients for endosymbionts, which in turn contribute to host nutrition acquisition, host utilization, stress resistance, and defense against natural enemies (21, 25). Aphid symbionts are classified as primary (obligate) and secondary (facultative) symbionts. Primary symbionts are essential for aphid development, whereas secondary symbionts vary in type and abundance depending on host and environmental conditions (26). However, the molecular mechanisms underlying their differential thermal tolerance remain unclear.

In this study, we investigated the green and red morphs of *M. persicae* under controlled laboratory conditions. Using 25 °C as the control, we exposed aphids to 30 °C and 35 °C for different durations. Transcriptome sequencing was performed to gene expression profiles and identify differences between the two morphs under heat stress. Differentially expressed genes (DEGs) were subjected to Gene Ontology (GO) and Kyoto Encyclopedia of Genes and Genomes (KEGG) enrichment analyses to identify candidate thermal tolerance-related genes and explore the molecular mechanisms underlying thermal adaptation. Additionally, 16S rDNA amplicon sequencing was employed to compare the microbial community composition and symbiont dynamics between color morphs under high-temperature stress. These results provide insights into the role of microbial symbiosis in thermal adaptation in *M. persicae*, offering a foundation for understanding aphid thermal tolerance and developing sustainable pest management strategies in the context of climate change.

## 2 Materials and methods

### 2.1 Aphid population collection

In July 2024, populations of the green peach aphid (*M. persicae*) were collected from tobacco fields under routine agricultural management in Qingzhou, Shandong Province, China (36°71′20″N, 118°46′64″E). Both the green and red color morphs of *M. persicae* were collected from the same tobacco leaf in Qingzhou. They were designated as QZ (green morph) and QZR (red morph), where the ‘R’ in QZR denotes the red color morph. For each morph, a single wingless adult aphid was isolated and individually reared to establish laboratory populations. In the laboratory, aphids were maintained on fresh leaves of the tobacco variety K326 under controlled conditions: 25 ± 1 °C temperature, 65 ± 1% relative humidity, and a 16:8 h light/dark photoperiod. Additionally, a green *M. persicae* population (JM), which had been continuously maintained in the laboratory for several generations and originally collected from tobacco fields in Jimo, Qingdao, Shandong Province, was used for third-generation transcriptome sequencing as a reference. Since the published *M. persicae* reference genome was based on data from 2016, third-generation transcriptome resequencing was performed in this study to obtain updated reference sequences.

### 2.2 Species identification

A random sample of 20–30 wingless adult aphids from both green and red morph populations of *M. persicae* was collected for DNA extraction according to the kit instructions (Tiangen Biotech, Beijing, China). The extracted DNA was then used for species identification based on the mitochondrial cytochrome oxidase I (COI) gene, amplified with the primers F: 5′-GGTCAACAAATCATAAAGATATTGG-3′ and R: 5′-TAAACTTCAGGGTGACCAAAAAATCA-3′. Sequencing results confirmed that all sampled individuals belonged to *M. persicae*, verifying the species identity of both color morphs.

### 2.3 Third-generation transcriptome aphid sample collection

A total of 100–120 wingless adult aphids from the JM population were collected for RNA extraction and third-generation transcriptome sequencing. The resulting full-length transcriptome data were used to construct a species-specific Unigene library, providing a transcript-level reference that eliminates the need for sequence assembly. This reference dataset serves as a robust foundation for subsequent analyses of gene expression and thermal stress responses.

### 2.4 Heat stress treatments

Physiological adaptation study: Fifty to sixty wingless adult aphids from both the QZ (green morph) and QZR (red morph) populations were selected and transferred onto individual tobacco plants. Each plant was enclosed with a transparent glass cover to prevent aphid escape. The plants were then placed in a controlled-environment chamber (light intensity: 2000 lux, photoperiod: 16L:8D, relative humidity: 65%) set at either 30 °C or 35 °C. Aphids were collected after exposure to the high-temperature treatments for 6, 12, and 24 h according to previous studies (27), with 25 °C serving as the control condition. Three biological replicates (a, b, c) were established for each time point, with 8-12 wingless adults per 1.5 mL centrifuge tube. Samples were immediately frozen in liquid nitrogen and stored at -80 °C for subsequent RNA sequencing analysis. The naming rules for transcriptome samples are as follows:0, 1, 2 indicate samples collected at 25 °C (control), 30 °C and 35 °C, respectively.A, B, C represent sampling times of 6 h, 12 h and 24 h.For example, QZ1A indicates that the sample from the QZ group was subjected to temperature at 30 °C for 6 hours.

Microbial reaction study: The experimental procedure was identical to that described in Section 2.4.1, except that the sampling intervals were set at 12, 24, and 48 h according to previous studies, respectively (28). The naming rules for microbiome samples are as follows:0, 1, 2 indicate samples collected at 25 °C (control), 30 °C and 35 °C, respectively.A, B, C represent sampling times of 12 h, 24 h and 48 h.For example, QZ1A indicates that the sample from the QZ group was subjected to temperature at 30 °C for 12 hours.

### 2.5 Transcriptome sequencing and data analysis

Third-generation transcriptome sequencing: Total RNA was extracted from aphid samples described in Section 2.3 using TRIzol reagent. Genomic DNA contamination was removed using DNase I (RNase-free). RNA integrity was verified by 1% agarose gel electrophoresis, and RNA quantity and purity were assessed using a NanoDrop spectrophotometer. cDNA synthesis was performed with the Iso-Seq Express 2.0 Kit, and library construction was completed using the Kinnex Full-Length RNA Kit. Sequencing was conducted on the PacBio Revio platform (29). Quality control of the third-generation sequencing data was performed, and subreads.bam files were processed using the CCS algorithm to generate Circular Consensus Sequences (CCS). Full-length non-chimeric (FLNC) sequences from the same transcript were clustered using a hierarchical n·log(n) algorithm to produce consensus sequences, which were subsequently polished to obtain high-quality consensus transcripts for downstream analyses.

Second-generation transcriptome sequencing: Aphid samples collected as described in Section 2.4.1 were used for second-generation transcriptome sequencing. Messenger RNA (mRNA) was enriched from total RNA using Oligo (dT) magnetic beads. cDNA libraries were constructed and sequenced on the Illumina NovaSeq 6000 platform using the PE150 strategy. Library concentration was quantified using a Qubit 2.0 Fluorometer to ensure sufficient yield. Strict quality control was applied to raw reads to remove adapters and low-quality sequences using Fastp (v0.23.1). Clean reads were then aligned to the polished consensus transcripts obtained from third-generation sequencing, which served as the reference transcriptome, to ensure accurate sequence mapping.

### 2.6 Principal component analysis

PCA was performed on the expected number of Fragments Per Kilobase of transcript sequence per Millions base pairs sequenced (FPKM) values of both green and red morph aphid samples to evaluate gene expression variation. The analysis was conducted using the ade4 package in R (v4.0.3) (30). Data were centralized by subtracting the mean, and principal components were extracted using the prcomp function. PCA scatter plots were visualized using ggplot2, and sample scores on the first two principal components (PC1 and PC2) were used to assess clustering and expression divergence between treatments.

### 2.7 Gene functional annotation and differential expression analysis

Functional annotation of unigenes was performed using BLASTX (E-value < 1e−5) against multiple databases, including Nr, Nt, Pfam, COG, Swiss-Prot, KEGG, and GO. GO enrichment analysis was conducted with GOseq (v1.10.0) based on the Wallenius non-central hypergeometric distribution, while KEGG pathway enrichment was performed using KOBAS (v3.0), with significant pathways defined by p < 0.05.

Transcript quantification was performed using Salmon, and differential expression analysis was conducted with DESeq2 (31). Normalized read counts were used to assess gene expression levels, expressed as |log_2_(Fold Change)|. Differentially expressed genes (DEGs) were identified based on q-value < 0.05 and |log_2_(Fold Change)|, providing expression profiles for subsequent analyses of color morph–specific and temperature-related responses.

### 2.8 Microbial sequencing and data analysis

Microbial sequencing: Total DNA was extracted from aphid samples described in Section 2.4.2. The 16S rDNA V4 region was amplified using primers 515F (5′-GTGCCAGCMGCCGCGGTAA-3′) and 806R (5′-GGACTACHVGGGTWTCTAAT-3′). PCR reactions were carried out in a 30 µL system containing 15 µL of Phusion High-Fidelity PCR Master Mix (New England Biolabs), 0.2 µM of each primer, and 10 ng of genomic DNA template. PCR cycling conditions were as follows: initial denaturation at 98 °C for 1 min; 30 cycles of 98 °C for 10 s, 50 °C for 30 s, and 72 °C for 30 s; followed by a final extension at 72 °C for 5 min. The resulting PCR products were purified and used to construct sequencing libraries with the NEBNext Ultra II DNA Library Prep Kit. Library quality and concentration were verified using Qubit fluorometry and quantitative PCR (qPCR). Libraries that passed quality control were sequenced on the Illumina NovaSeq platform (250 bp paired-end reads) (Novogene Co., Ltd., Beijing, China).

Bacterial community characterization: Raw sequencing data were demultiplexed based on barcode and primer sequences. After trimming barcodes and primers, paired-end reads were merged using FLASH (v1.2.11). Quality filtering was performed with Fastp to obtain high-quality Clean Tags, which were then checked for chimeras against reference databases using Usearch, producing Effective Tags. Denoising and sequence variant inference were performed using the DADA2 module in QIIME2, and low-abundance sequences (counts < 5) were removed (32, 33). This generated the final Amplicon Sequence Variants (ASVs) and corresponding feature tables (34). Data were rarefied to 97% sequencing depth using the feature-table rarefy tool in QIIME2. Taxonomic classification of ASVs was conducted using the classify-sklearn module against a reference database. Relative abundance of microbial taxa was normalized using the decostand function in the vegan R package. Alpha diversity indices, including Shannon and Chao1, were calculated using the diversity function in R (v3.4.3). Differences in alpha diversity and community composition between the green and red morphs of *M. persicae* were subsequently analyzed and compared.

## 3 Results

### 3.1 Sequencing data quality

To investigate the transcriptional responses of green (QZ) and red (QZR) morphs of Myzus persicae to temperature stress, we performed RNA-seq on samples exposed to three temperatures (25 °C, 30 °C, and 35 °C) for varying durations (6 h, 12 h, and 24 h for one set; 12 h, 24 h, and 48 h for another set). The sequencing data quality was assessed to ensure reliability for downstream analyses. The PacBio third-generation sequencing produced 6.31 Gb of HiFi data, with all reads above Q20 (**Supplementary Table 1**). For Illumina sequencing, 1104.34 Gb of raw data and 1078.18 Gb of clean data were obtained after filtering. The QZ and QZR samples yielded 38.0-66.0 Mbp of clean reads, with Q30 values between 95.69% and 97.76% (**Supplementary Table 1**). After removing two low-quality samples, the remaining libraries showed 64.82%-77.51% mapping rates to the reference genome (**Supplementary Table 2**). For the additional set of samples treated for 12 h, 24 h, and 48 h, the GC content of valid reads ranged from 46.68% to 54.79%, with all samples showing Q20 > 98% and Q30 > 93% (**Supplementary Table 3**), confirming high sequencing accuracy and data reliability. FPKM values for the different samples in **Supplementary Table 4**.

### 3.2 Physiological responses of two color morphs of *Myzus persicae* under heat stress: principal component analysis

**Figure 1:**
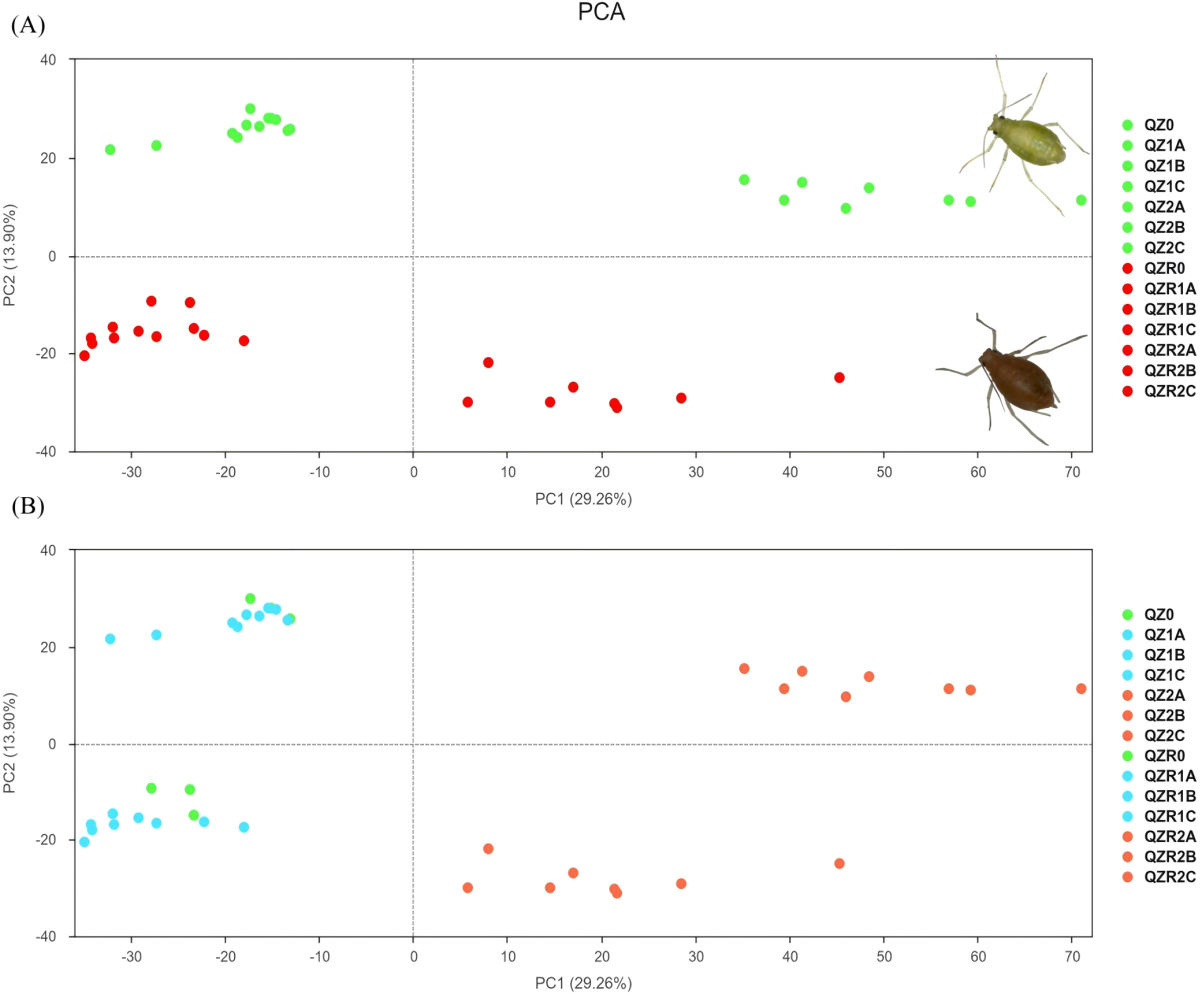
PCA analysis of gene expression among different temperature and time treatments in green and red morphs of *M. persicae* (|log_2(_Fold Change)|≥1 and q-value <0.05). **(A)** is labeled and described to illustrate the clustering of samples based on body color (red vs. green morphs). **(B)** is labeled and described to illustrate the clustering of samples based on temperature treatment. PCA analysis of gene expression among different time treatments in green and red morphs of *M. persicae* could be found in **Supplementary Figure 1**.

PCA was conducted to examine transcriptomic variation between the green and red morphs of *M. persicae* under different temperature and time treatments (**Figure 1** and **Supplementary Figure 1**). The results showed that PC1 effectively separated the 35 °C samples of both color morphs from those at 25 °C and 30 °C, with 35 °C samples clustering on the right side of the plot. PC2 distinguished between the green and red morphs, with red morph samples distributed toward the lower region and green morph samples toward the upper region of the PCA plot.

### 3.3 Physiological responses of two color morphs of *Myzus persicae* under heat stress: differentially expressed genes

**Figure 2:**
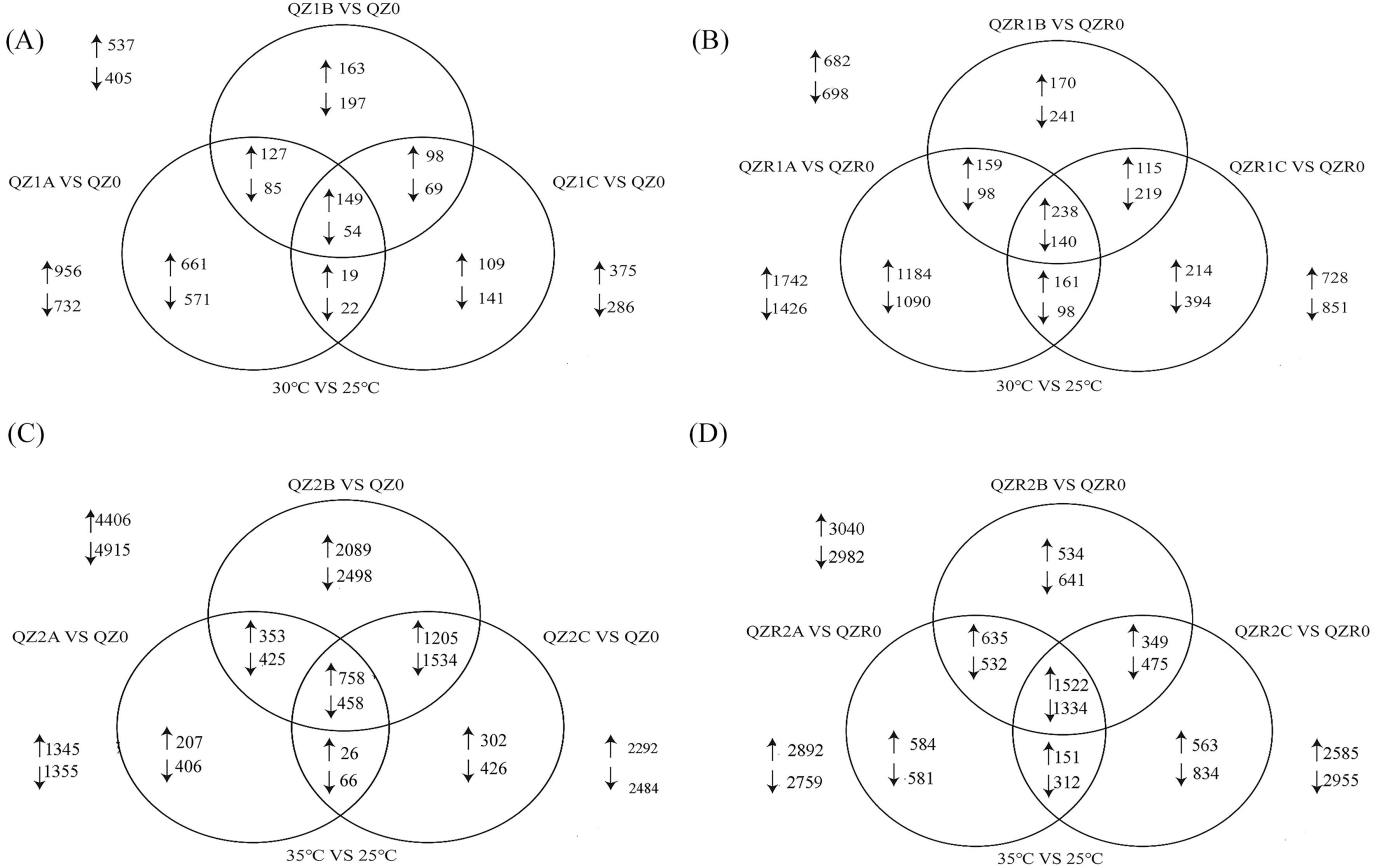
Venn diagrams of DEGs in green and red morphs of *M. persicae* under different treatments. Panel A and Panel B show the numbers of DEGs under 30°C with different time treatment in the green **(A)** and red **(B)***M. persicae* morphs, respectively. Panel C and Panel D show the numbers of DEGs under 35°C with different time treatment in the green **(C)** and red **(D)** morphs (|log_2_(Fold Change)| ≥ 1 and q-value <0.05). An upward arrow indicates up-regulated expression, while a downward arrow indicates down-regulated expression.

Venn diagrams (**Figure 2**) illustrate the overlap and unique numbers of DEGs across temperature treatments, revealing that numerous genes exhibited distinct expression patterns under different thermal conditions (|log_2_(Fold Change)| ≥ 1 and q-value < 0.05). For the green morph, 203 DEGs were detected after exposure to 30 °C for 6, 12, and 24 h, including 149 up-regulated and 54 down-regulated genes. The number of up-regulated genes gradually decreased with prolonged exposure. Under 35 °C, 758 genes were up-regulated, substantially higher than at 30 °C, indicating that more genes were activated to cope with elevated thermal stress; the up-regulated gene count first increased and then declined over time. For the red morph, 378 DEGs were identified at 30 °C (238 up-regulated, 140 down-regulated), a higher total than in the green morph. The number of up-regulated genes showed a decreasing-then-increasing trend over time. Under 35 °C, 1522 genes were up-regulated, more than twice that of the green morph, showing a similar temporal pattern (initial rise then decline).

### 3.4 Physiological responses of two color morphs of *Myzus persicae* under heat stress: GO and KEGG functional enrichment analysis of DEGs

**Figure 3:**
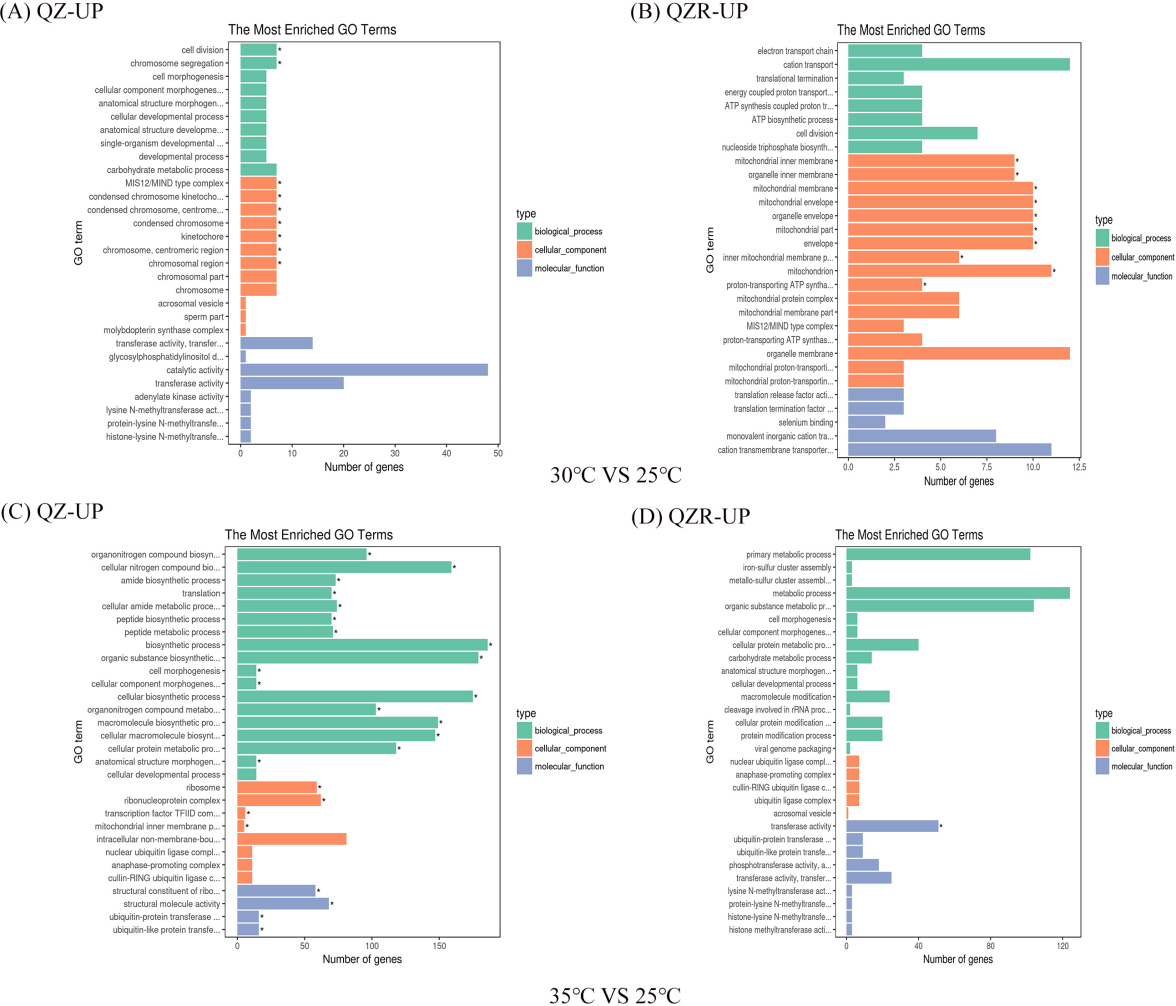
GO enrichment analysis of shared up-regulated DEGs in the green and red morph of *M. persicae*. Panel A and Panel B show the GO enrichment analysis of shared up-regulated DEGs under the 30°C treatment in the green **(A)** and red **(B)***M. persicae* morphs, respectively. Panel C and Panel D show the GO enrichment analysis of shared up-regulated DEGs under the 35°C treatment in the green **(C)** and red **(D)** morphs(|log_2_(Fold Change)| ≥ 1 and q-value <0.05). The top 30 enriched GO terms are displayed. Asterisks indicate significant enrichment at p < 0.05.

Functional enrichment analyses were conducted to better interpret the biological significance and regulatory roles of DEGs in different color morphs of *M. persicae*. GO enrichment was performed across the three major categories—Biological Process (BP), Molecular Function (MF), and Cellular Component (CC)—and the top 30 enriched GO terms were visualized (**Figure 3** and **Supplementary Figure 2**).

For the green morph, DEGs at 30 °C were significantly enriched in the CC term, particularly in chromosome-related functions. At 35 °C, more BP related genes were activated than at 30 °C. Up-regulated DEGs were particularly enriched in biosynthetic and metabolic processes, and translation, while several glucosyltransferase related genes were down-regulated.

In contrast, for the red morph, DEGs at 30 °C were enriched in metabolic pathways, with up-regulated genes predominantly associated with mitochondrion, especially membrane composition, and down-regulated genes fewer in number. The most represented biological processes included transport and localization. At 35 °C, enrichment was mainly observed in oxidoreductase activity within Molecular Function, but overall metabolic pathway enrichment was lower than at 30 °C—opposite to the pattern seen in the green morph. These findings suggest that the two color morphs employ distinct functional gene regulation mechanisms under heat stress.

To further explore the biological pathways involved, KEGG enrichment analysis was performed. The top 20 enriched pathways were visualized as bubble plots (**Supplementary Figures 3**, **4**). In the green morph, at 30 °C vs. 25 °C, the most significantly enriched pathway was the spliceosome, followed by protein processing in the endoplasmic reticulum and endocytosis. At 35 °C, the ribosome pathway showed the highest enrichment, indicating enhanced protein synthesis activity.

For the red morph, the overall enrichment significance of the top 20 pathways at 30 °C was lower than that in the green morph, although eight pathways were shared between the two morphs. Differences in enrichment magnitude and expression intensity indicate divergent regulatory strategies under thermal stress. At 35 °C, DEGs in the red morph were primarily enriched in protein processing in the *endoplasmic reticulum* and *endocytosis*, rather than the ribosome pathway. Several genes were also involved in carbohydrate and amino acid metabolism.

### 3.5 Physiological responses of two color morphs of *Myzus persicae* under heat stress: expression analysis of key heat-responsive genes

**Figure 4:**
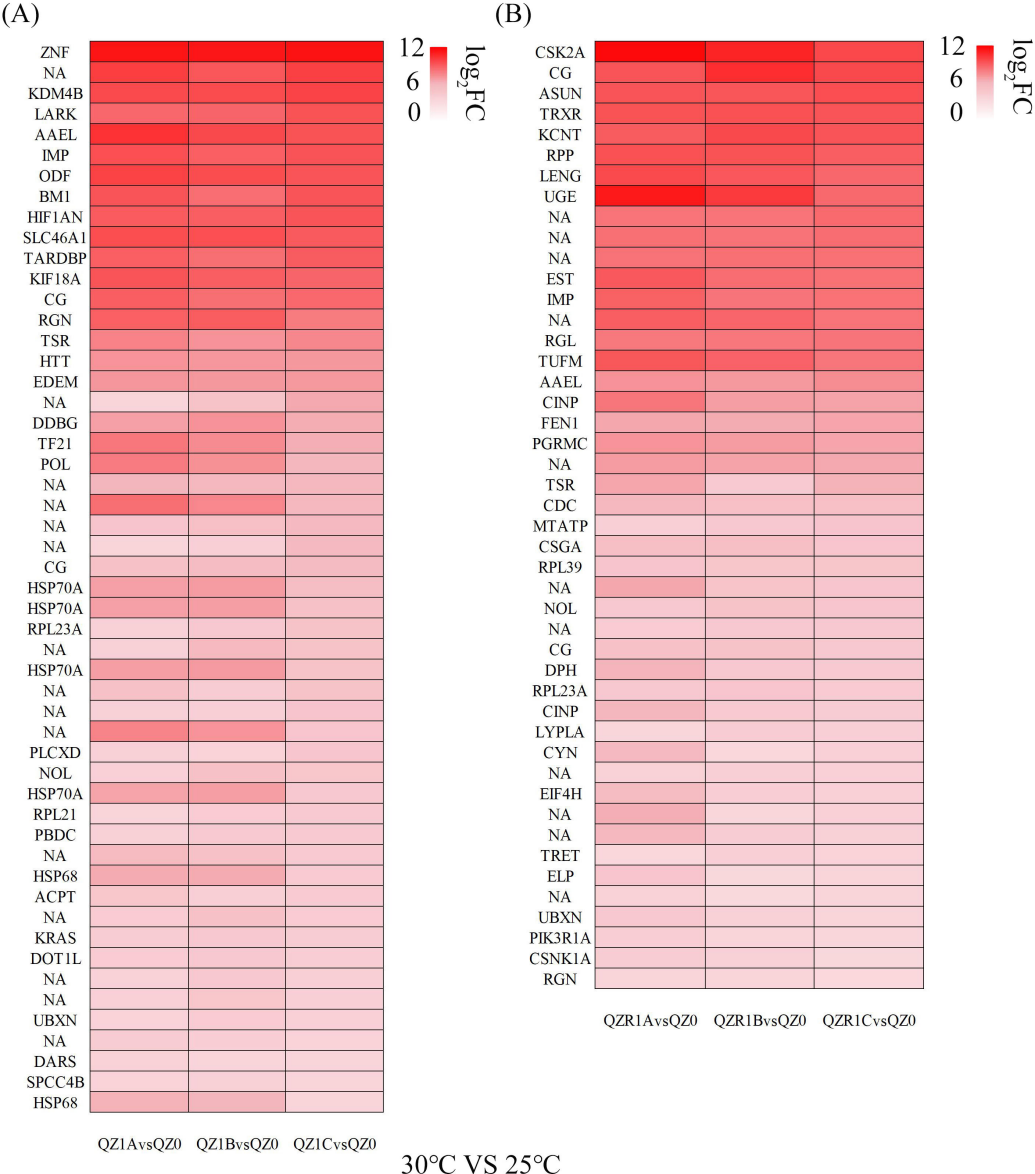
Heatmap of shared up-regulated heat tolerance related genes under 30 °C with different time treatment in green **(A)** and red **(B)** morphs of *M. persicae* (|log_2_(Fold Change)| ≥ 2 and q-value <0.05). Rows without labels correspond to transcripts with no assigned gene symbol (NA) in the database. Each column represents the mean expression value of three biological replicates for the indicated condition.

**Figure 5:**
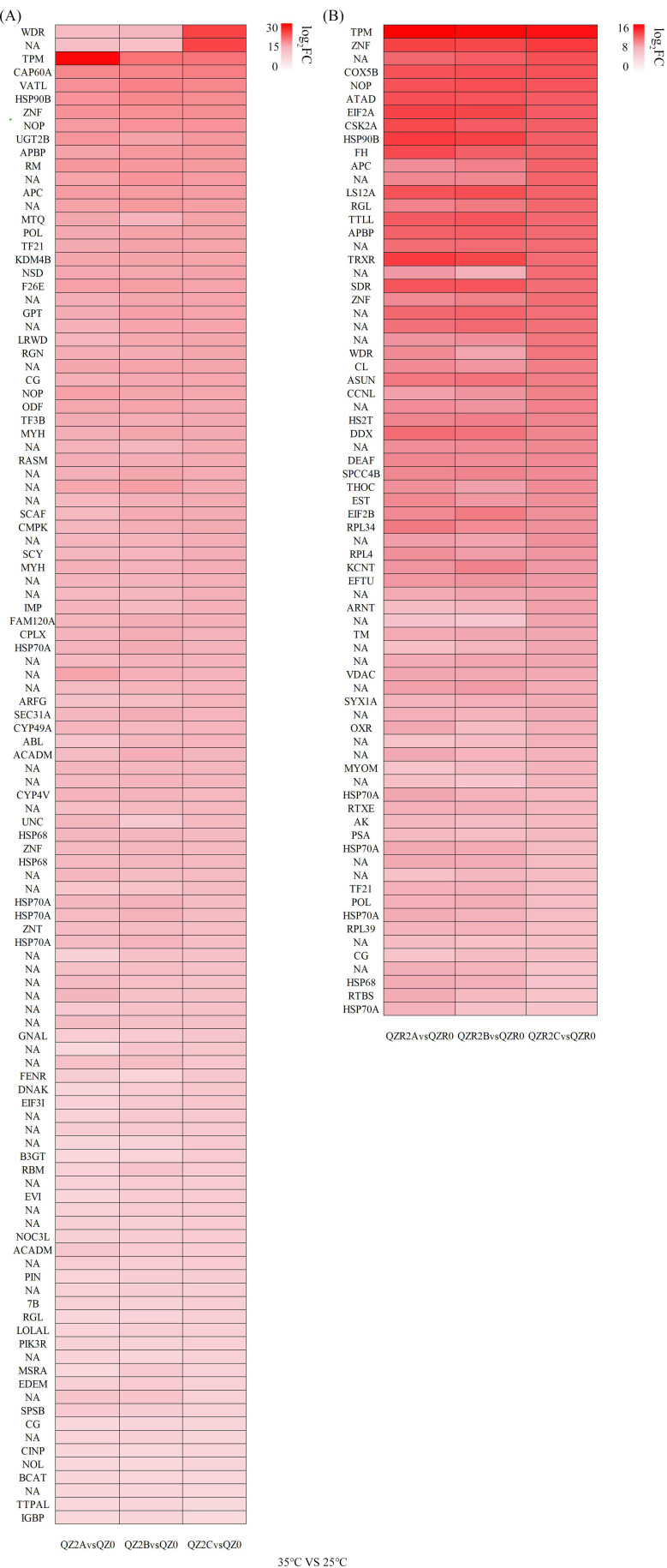
Heatmap of shared up-regulated heat tolerance related genes under 35 °C with different time treatment in green **(A)** and red **(B)** morphs of *M. persicae* (|log_2_(Fold Change)| ≥ 4 and q-value <0.05). Rows without labels correspond to transcripts with no assigned gene symbol (NA) in the database. Each column represents the mean expression value of three biological replicates for the indicated condition.

Heatmaps based on |log_2_(Fold Change)|≥ 2/4 and q-value < 0.05 revealed genes regulated under varying temperature stress (**Figures 4**, **5**). After exposure to different high-temperature stresses, several HSP family genes were up-regulated in the green morph, with *Hsp70* (*Hsp70A*) and *Hsp68* showing consistently high expression levels. As the temperature increased, *Hsp90B* was additionally up-regulated, exhibiting a more than 20-fold increase at 6 h post-exposure to 35°C, which later declined to approximately 12-fold with prolonged stress, indicating a coordinated heat stress–response mechanism. In contrast, the red morph showed no significant upregulation of HSP genes at 30°C. However, exposure to severe heat stress (35 °C) triggered strong activation of *Hsp70A*, *Hsp68*, and *Hsp90B* in both morphs, with the red morph exhibiting a delayed but more pronounced induction of *Hsp*90B over time.

Furthermore, under 30°C stress, transcription factors (TFs) and zinc finger proteins (ZNFs) were only activated in green morph. After exposure to 35°C, high expression of TFs and ZNFs was observed in red morph, and the green morph maintained consistently high expression levels of these genes across treatments. We also found that after exposure to 30°C or 35°C, RPL family ribosomal protein genes—previously reported to function as stabilizers of the cellular translation system in insect heat tolerance—were up-regulated in both color morphs.

At 35°C, several CYP family genes were markedly upregulated in the green morph (|log_2_(Fold Change)| ≥ 4), whereas expression levels in the red morph were relatively lower (|log_2_(Fold Change)| ≥ 2) (**Supplementary Table 6**), indicating a substantial increase in detoxification enzyme abundance in the green morph. Additionally, the UGT family gene *Ugt2B* showed high expression levels in the green morph under 35°C treatment.

### 3.6 Microbial responses to heat stress in two color morphs of *M. persicae:* Microbial composition and abundance

**Figure 6:**
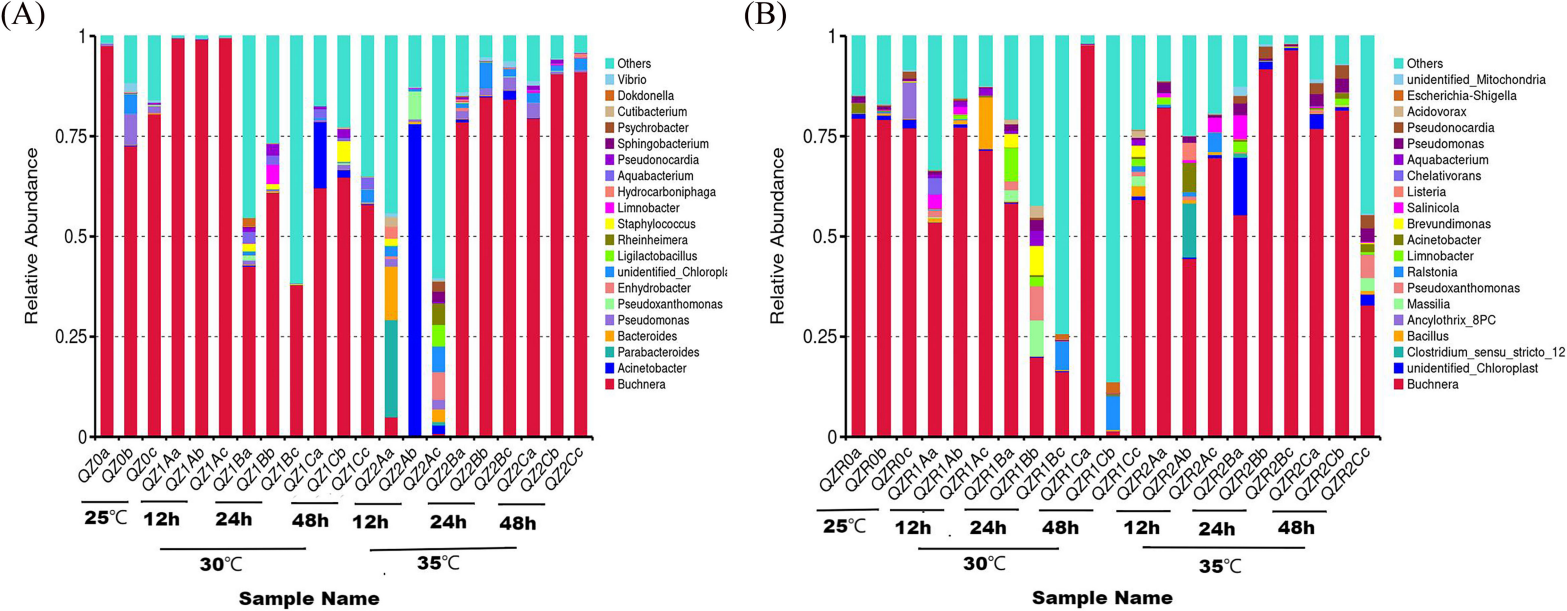
Relative abundance of bacterial taxa in the green **(A)** and red **(B)** morphs of *M. persicae* under different temperatures and sampling times. The colors in the bar plots represent different taxonomic groups; relative abundance is defined as the proportion (or percentage) of sequences assigned to a specific taxonomic group relative to all sequences obtained from a sample.

**Figure 7:**
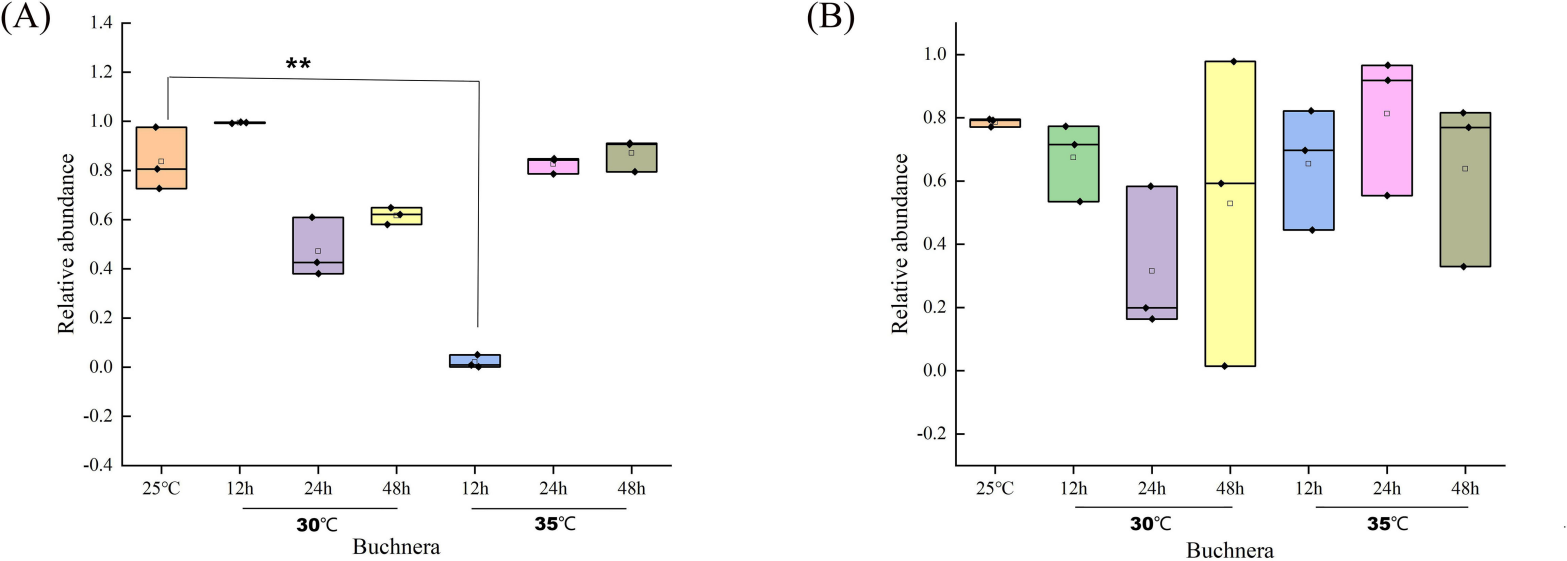
Variation in *Buchnera* abundance across temperatures and sampling times in green **(A)** and red **(B)** morphs of *M. persicae.* Data are presented as mean ± SEM. Significant differences (compared to the 25 °C) are marked with asterisks (* p < 0.05, ** p < 0.01, *** p < 0.001).

The top 20 bacterial genera were identified in both morphs after heat stress. *Buchnera aphidicola* was consistently dominant (**Figure 6**). In the green morph, *Buchnera* accounted for > 75% at 25 °C, peaked at 30 °C (12 h), and declined thereafter; at 35 °C, its abundance significantly dropped but recovered gradually over time. In the red morph, *Buchnera* remained relatively stable at all temperature (**Figure 7**).

### 3.7 Microbial responses to heat stress in two color morphs of *M. persicae:* alpha diversity analysis

All samples showed coverage values above 0.99, indicating sufficient sequencing depth. The mean Chao1 index was 335.25 (range: 55.82-679.22), suggesting high bacterial richness. The red morph exhibited the highest Chao1 value (679.22) at 30 °C after 48 h, while the green morph showed the highest Shannon diversity (5.70) at 35 °C after 12 h and the lowest (0.08) at 30 °C after 12 h (**Supplementary Table 7**).

## 4 Discussion

### 4.1 Differential gene responses of green and red morphs to heat stress

The present study demonstrates that the green and red morphs of *M. persicae* exhibit distinct transcriptional and microbial responses under heat stress, reflecting divergent thermal tolerance strategies. PCA revealed that temperature stress had a stronger influence on gene expression than body color differences, and the transcriptional response was most pronounced at 35 °C, suggesting that heat stress substantially alters the physiological state of *M. persicae* and even a modest temperature increase can trigger substantial physiological reprogramming.

The scale and expression patterns of temperature-responsive genes between the two morphs suggest that they adopt distinct molecular pathways to cope with thermal stress. Compared with the green morph, the red morph activated a broader set of genes under heat stress, potentially involving more complex physiological and biochemical processes for thermal adaptation. However, the green morph exhibited earlier and stronger transcriptional activation of heat-responsive genes, particularly those related to HSPs, RPLs, ZNFs, TFs and detoxification metabolism. These genes are commonly associated with stress resistance, indicating an active host gene–driven response to heat stress. In contrast, the red morph showed delayed and more focused induction of HSP genes mainly under severe heat stress, suggesting a more conservative heat tolerance strategy relying on physiological buffering and symbiont stability. At the same time, the green morph exhibited significant down-regulation of oxidative stress-related genes and limited activation of HSPs, suggesting a more conservative metabolic adjustment to minimize energy expenditure under prolonged thermal exposure.

Such polymorphic differences in heat response are consistent with prior studies linking insect color morphs to environmental adaptability. Darker body coloration, often associated with higher melanin content, can enhance heat absorption and facilitate thermal stability (24). The red morph’s higher melanin levels may enable more efficient heat tolerance, reflected in its transcriptional upregulation of stress-response genes. In contrast, the green morph, rich in carotenoids, may rely on antioxidant pathways to resist oxidative damage induced by moderate temperature elevations. Together, these mechanisms illustrate the evolutionarily diversified strategies of *M. persicae* morphs for surviving in variable thermal environments.

### 4.2 Body color-linked mechanisms of thermal adaptation

Body coloration of *M. persicae* strongly influences insect thermal regulation and stress tolerance. The color-based traits shape distinct thermal strategies: the green morph activates strong and fast HSP, detoxification and heat-related responses, whereas the red morph maintains moderate and slow adjustment and protection (35, 36). The slower transcriptional activation observed in red morphs under heat exposure may result from their higher carotenoid content and symbiont-mediated buffering capacity (37). Carotenoids provide antioxidant protection and reduce cellular damage, diminishing the need for rapid induction of heat shock and detoxification genes.

Pigment-based traits exhibit different patterns in other insects. Melanic *Harmonia axyridis* beetles warm faster and survive better in cool habitats, while lighter morphs are favored in warmer climates (38). *Drosophila melanogaster* populations show darker coloration in colder regions, reflecting adaptive thermal melanism (27). However, red morphs of *A. pisum* exhibit stronger heat-shock responses than green morphs (24). This might be explained by that *M. persicae* and *A. pisum* differ substantially in their genomic and metabolic backgrounds, including the horizontally transferred carotenoid biosynthesis genes responsible for pigmentation (21). The distinct integration sites and regulation of these fungal-origin carotenoid genes can lead to variation in pigment composition and downstream physiological functions. In *M. persicae*, red pigmentation may be linked to oxidative stress signaling or host defense responses, while in *A. pisum*, red pigments are more tightly associated with enhanced antioxidant and heat-shock pathways (21).

Together, these findings suggest that pigmentation in *M. persicae* is associated with differential expression of genes involved in biochemical protection and thermal regulation, which may contribute to the persistence of color morphs across variable thermal environments.

### 4.3 Heat shock proteins and detoxification systems in thermal adaptation

HSPs play a central role in maintaining protein stability, refolding denatured proteins, and preventing aggregation under stress (39, 40). In our study, several HSP genes, including *Hsp70*, *Hsp90*, and small HSPs, were significantly up-regulated at 35°C, especially in the green morph. Similar findings have been reported in *L. hesperus* and *G. molesta*, where high temperatures induced strong HSP expression to preserve cellular homeostasis (6, 7). The robust up-regulation of HSPs implies superior protein protection capacity and rapid heat-shock response, which may partly explain the aphid’s sensitivity to heat.

In addition to HSPs, detoxification-related genes, including *Cyp4V, Cyp49A, Cyp4C, Cyp6A* and *Ugt2B* were up-regulated under heat stress. These enzymes might help alleviate heat-induced oxidative stress by neutralizing reactive oxygen species and repairing damaged bio-molecules (41). In addition to their roles in mitigating heat-induced oxidative stress, cytochrome P450s and UDP-glucuronosyltransferases are key detoxification enzymes involved in xenobiotic metabolism and endogenous compound turnover, processes that may be broadly altered under thermal stress. In this study, several members of the *Cyp4*, *Cyp6*, and *Ugt2B* families were prominently upregulated, suggesting potential functional differentiation among gene families in supporting metabolic homeostasis and stress tolerance.

### 4.4 Transcriptional and post-translational regulation underlying heat adaptation

High-temperature stress triggered extensive transcriptional and translational reprogramming in *M. persicae*. GO and KEGG enrichment revealed genes related to transcriptional regulation, protein folding, and so on, indicating integrated mechanisms for maintaining protein homeostasis under heat exposure.

Transcription factors (TFs) including zinc finger (ZNF) proteins were strongly upregulated, suggesting that *M. persicae* activates a hierarchical gene regulatory network during heat stress (42, 43). The green morph showed earlier activation of TFs under 30°C, indicating anticipatory transcriptional regulation, whereas the red morph displayed delayed but stronger transcriptional responses at 35°C.

At the post-translational level, pathways such as “protein processing in endoplasmic reticulum” and “spliceosome” were significantly enriched, reflecting intensified protein quality control. The red morph showed stronger activation of endoplasmic reticulum-related and RPL genes, suggesting enhanced translational stabilization and recovery from heat stress.

Overall, *M. persicae* exhibits a dual-layered regulatory strategy: the green morph relies on early transcriptional modulation, while the red morph emphasizes rapid protein repair and folding to restore cellular function. These morph-specific patterns illustrate distinct molecular mechanisms of aphid thermal adaptation. These results suggest that *M. persicae* mobilizes a complex transcriptional network to orchestrate thermotolerance, integrating stress signaling with metabolic regulation to sustain survival under prolonged heat exposure.

### 4.5 Microbial symbionts as modulators of host thermal tolerance

The 16S rDNA sequencing results further highlight the potential role of microbial symbionts in modulating the thermal responses of *M. persicae* (9–11). The dominant primary symbiont *Buchnera* exhibited morph-specific abundance dynamics under heat stress-declining sharply in the green morph but remaining stable in the red morph. As *Buchnera* is essential for amino acid synthesis and nutrient supply, its stability is closely linked to host fitness. Previous studies have shown that heat stress can reduce *Buchnera* density, compromising host fecundity and survival (3). The maintenance of *Buchnera* stability in the red morph suggests a stronger host-symbiont interaction and may represent a critical factor in its enhanced heat resistance. These results indicate that *Buchnera* dynamics differ between morphs and contribute to their distinct thermal tolerance capacities.

### 4.6 Integrative perspective on thermal adaptation mechanisms

Integrating transcriptomic and microbiome data, this study suggests that the thermal tolerance of *M. persicae* is achieved through a multi-layered adaptive system that involves both intrinsic molecular regulation and symbiont-mediated modulation. The red morph appears to rely on active stress responses, including HSP induction, detoxification, and symbiont stability, while the green morph may adopt a more passive strategy, prioritizing metabolic suppression and energy conservation.

Such morph-specific strategies provide adaptive flexibility, allowing *M. persicae* populations to persist under variable temperature conditions. These findings align with the broader understanding that insect adaptation to thermal stress involves both genetic plasticity and symbiotic cooperation (44). The integration of host and microbial responses thus represents a fundamental evolutionary mechanism enabling aphids to maintain ecological dominance under global warming.

## 5 Conclusion

In conclusion, this study reveals the distinct molecular and microbial strategies underlying thermal adaptation in the green and red morphs of *M. persicae*. Transcriptomic and 16S rDNA analyses demonstrate that both morphs activate stress-related genes under heat stress, but with different regulatory intensities. The green morph displayed stronger induction of heat adaptation-related genes, indicating an active stress-defense mechanism. In contrast, the red morph adopted a more conservative response by moderating energetic processes, coupled with greater stability of its primary symbiont, *Buchnera aphidicola*. This symbiont stability appears to be a key modulator contributing to the red morph’s higher heat tolerance. Overall, thermal resilience in *M. persicae* emerges from the integrated coordination between host gene regulation and symbiont dynamics. These findings provide a mechanistic basis for predicting morph-specific population dynamics of *M. persicae* under climate warming and offer valuable insights for developing climate-adaptive pest management strategies.

## Data Availability

The original contributions presented in the study are included in the article/**Supplementary Material**. Further inquiries can be directed to the corresponding authors.
