# Supplementary material for: Integrative transcriptomic and microbiome analyses reveal thermal adaptation mechanisms in green and red color morphs of Myzus persicae (Hemiptera: Aphididae)
Source: Front Insect Sci. 2026 Apr 17;6:1780864. doi: 10.3389/finsc.2026.1780864 (PMC13133051; doi:10.3389/finsc.2026.1780864)
Supplement: Supplementary file 1 [file DataSheet1.pdf]

Supplementary Figures

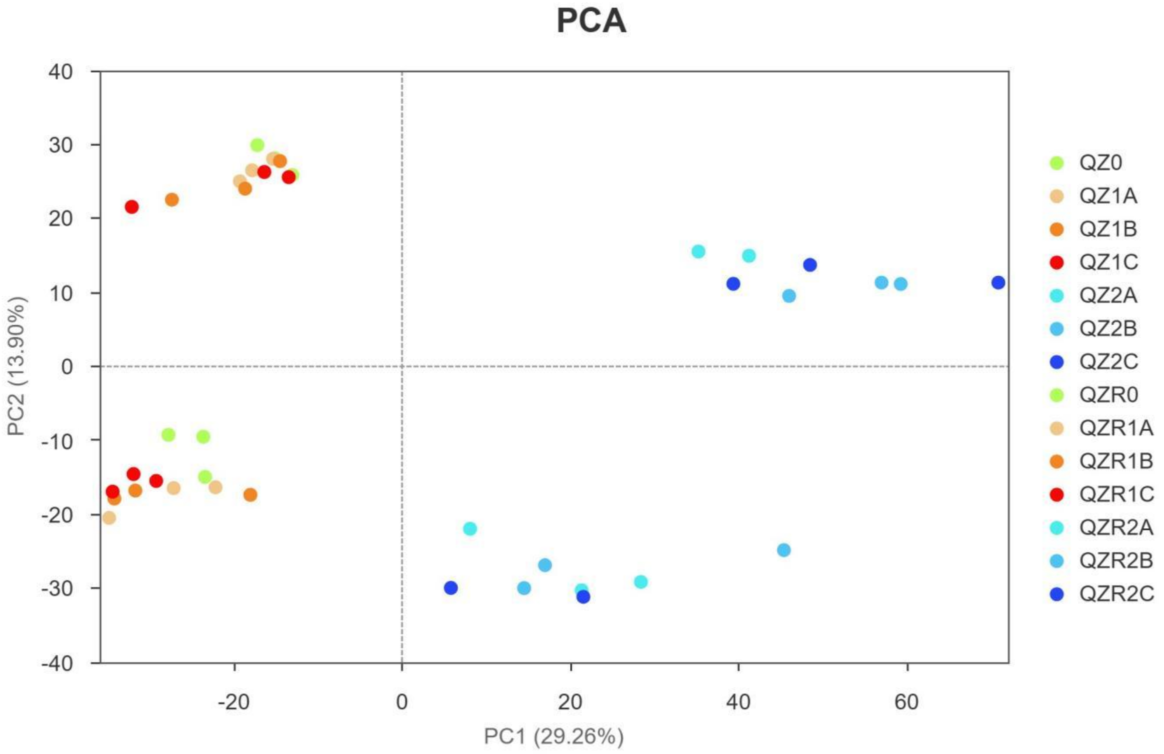

Supplementary Figure 1. PCA analysis of gene expression in green and red forms of *M.persicae* under different temperature and time treatments marked by different sampling times ($|\log_2(\text{Fold Change})| \geq 1$ and q value < 0.05).

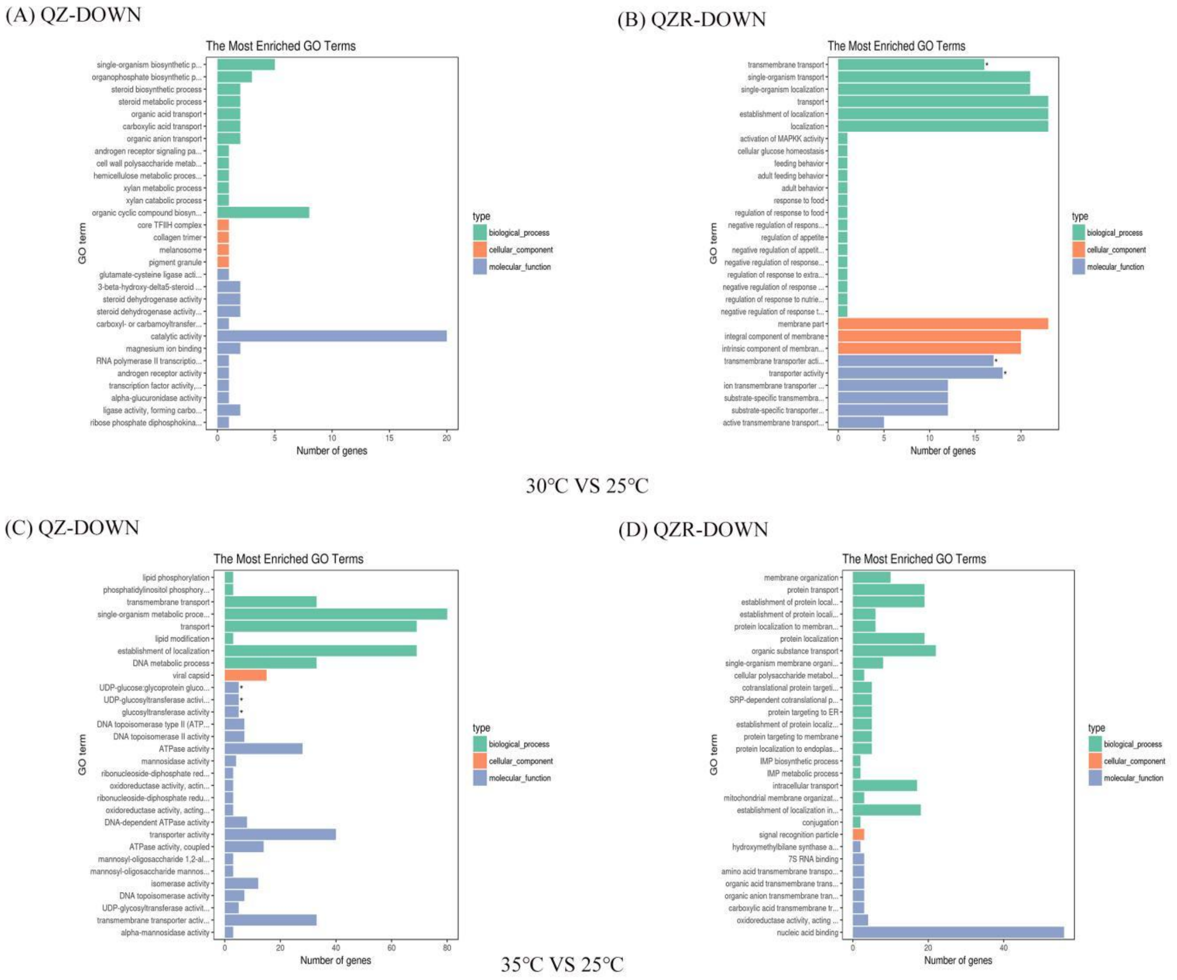

Supplementary Figure 2. GO enrichment of shared down-regulated DEGs ($|\log_2(\text{Fold Change})| \geq 1$ and q-value < 0.05) in the green and red morph of *M.persicae*. Panel A and Panel B show the GO enrichment analysis of shared down-regulated DEGs under the 30°C treatment in the green (A) and red (B) *M.persicae* morphs, respectively. Panel C and Panel D show the GO enrichment analysis of shared down-regulated DEGs under the 35°C treatment in the green (C) and red (D) morphs. The top 30 enriched GO terms are displayed. Asterisks indicate significant enrichment at$p < 0.05$.

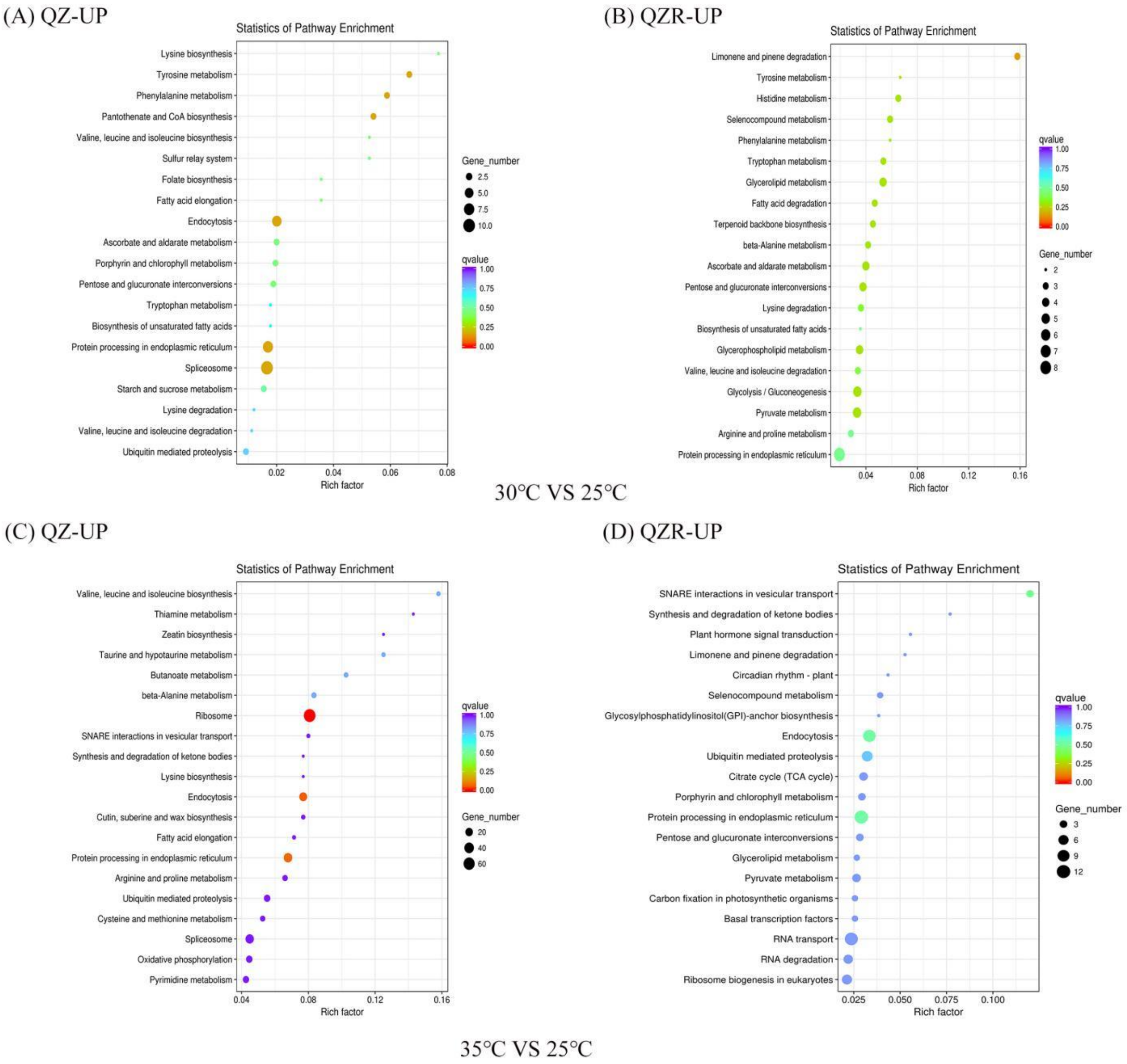

Supplementary Figure 3. KEGG enrichment scatter plots of shared up-regulated DEGs (|$\log_2$(Fold Change)| ≥ 1 and q-value < 0.05) in green and red morph of *M.persicae*. Panel A and Panel B show the KEGG enrichment analysis of shared up-regulated DEGs under the 30°C treatment in the green (A) and red (B) *M.persicae* morphs, respectively. Panel C and Panel D show the enrichment analysis of shared up-regulated DEGs under the 35°C treatment in the green (C) and red (D) morphs. The top 20 KEGG pathways are shown.

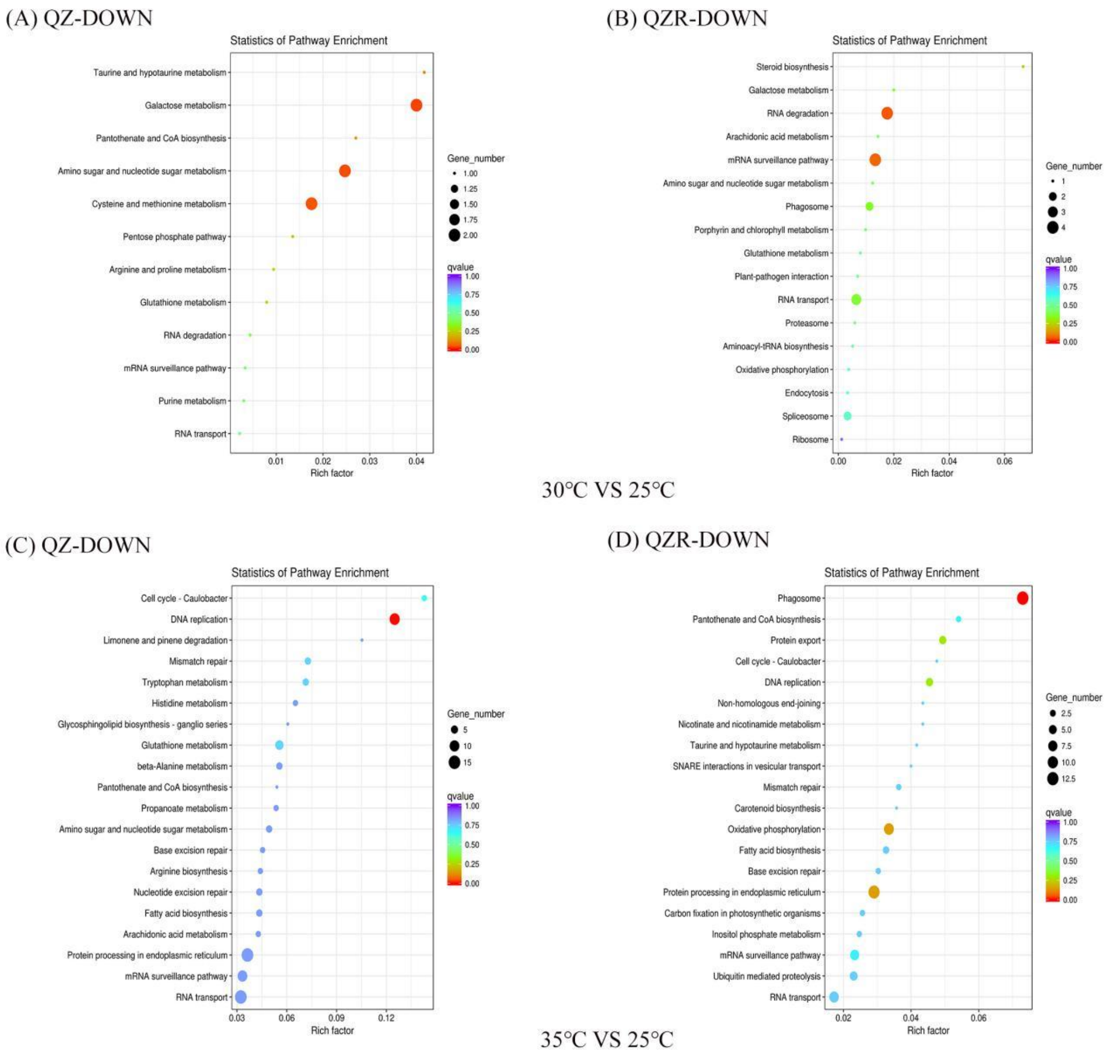

Supplementary Figure 4. KEGG enrichment scatter plots of shared down-regulated DEGs ($|\log_2(\text{Fold Change})| \geq 1$ and q-value < 0.05) in green and red morph of *M.persicae*. Panel A and Panel B show the KEGG enrichment analysis of down-regulated DEGs under the 30°C treatment in the green (A) and red (B) *M.persicae* morphs, respectively. Panel C and Panel D show the down-regulated DEGs under the 35°C treatment in the green (C) and red (D) morphs. The top 20 enriched KEGG pathways are shown.

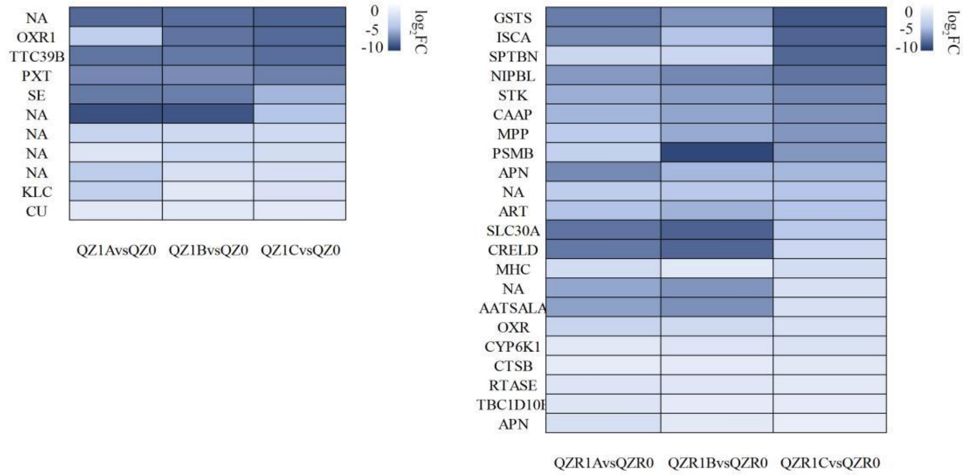

30°C VS 25°C

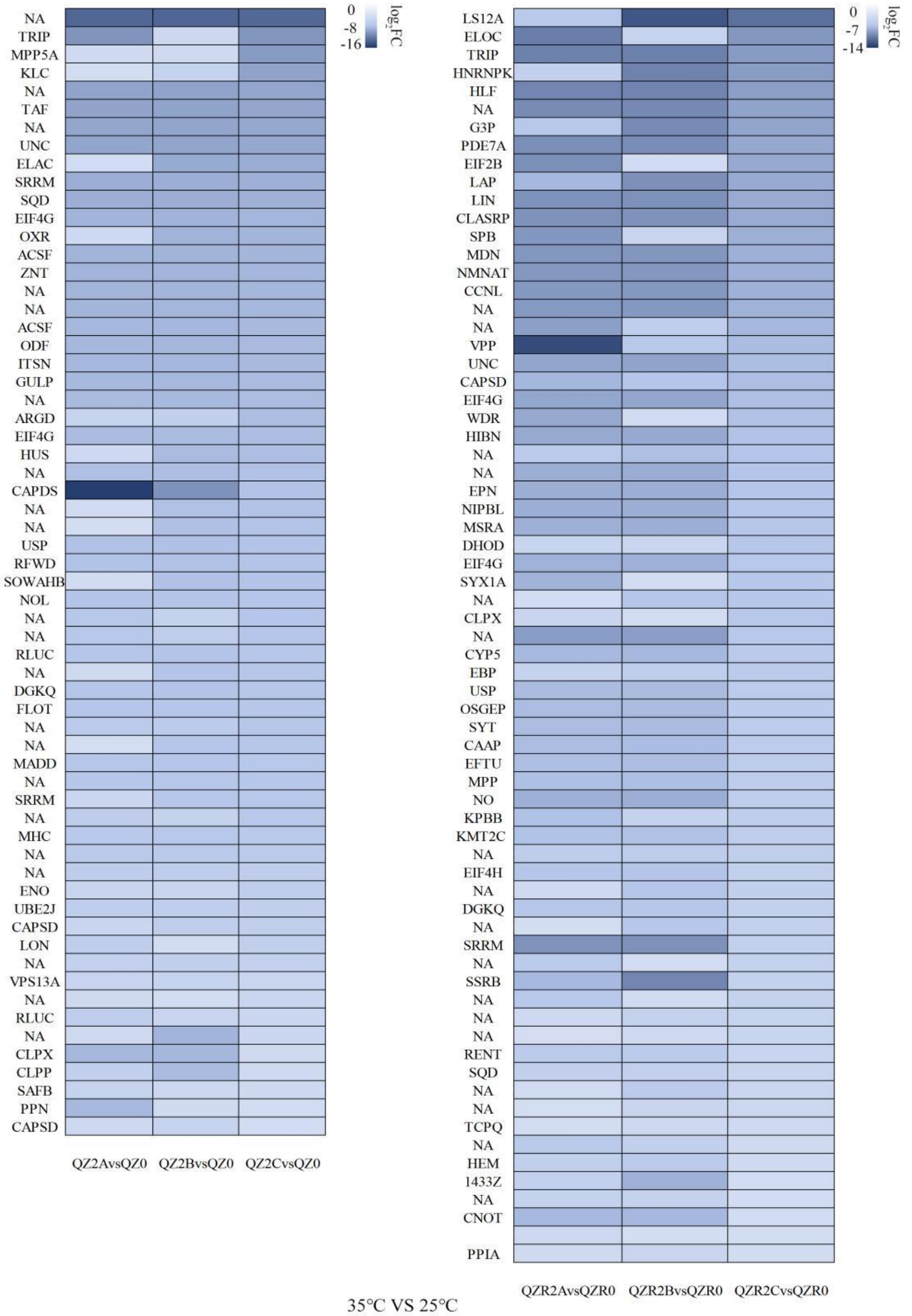

35°C VS 25°C

Supplementary Figure 5. Heatmap of downregulated heat tolerance-related genes in green and red morphs of *M.persicae*. Panel A and Panel B show the down-regulated DEGs with $|\log_2(\text{Fold Change})| \geq 2$ and q-value <0.05 under the 30°C treatment in the green (A) and red (B) *M.persicae* morphs, respectively. Panel C and Panel D show the down-regulated DEGs with $|\log_2(\text{Fold Change})| \geq 4$ and q-value <0.05 under the 35°C treatment in the green (C) and red (D) morphs. NA denotes transcripts lacking an assigned gene symbol. Each column represents the mean expression value of three biological replicates for the indicated condition.
